# Peri-operative chemotherapy with or without bevacizumab in operable oesophagogastric adenocarcinoma (UK Medical Research Council ST03): primary analysis results of a multicentre, open-label, randomised phase 2–3 trial

**DOI:** 10.1016/S1470-2045(17)30043-8

**Published:** 2017-03

**Authors:** David Cunningham, Sally P Stenning, Elizabeth C Smyth, Alicia F Okines, William H Allum, Sam Rowley, Laura Stevenson, Heike I Grabsch, Derek Alderson, Thomas Crosby, S Michael Griffin, Wasat Mansoor, Fareeda Y Coxon, Stephen J Falk, Suzanne Darby, Kate A Sumpter, Jane M Blazeby, Ruth E Langley

**Affiliations:** a Department of Oncology, Royal Marsden NHS Foundation Trust, London, UK; b Medical Research Council Clinical Trials Unit, University College London, London, UK; c Department of Surgery, Royal Marsden NHS Foundation Trust, London, UK; d Section of Pathology and Tumour Biology, Leeds Institute of Cancer and Pathology, University of Leeds, Leeds, UK; e Department of Pathology, GROW School for Oncology and Developmental Biology, Maastricht University Medical Center, Maastricht, Netherlands; f Department of Surgery, Queen Elizabeth Hospital, Birmingham, UK; g Department of Clinical Oncology, Velindre Hospital, Cardiff, UK; h Northern Oesophagogastric Cancer Unit, Royal Victoria Infirmary, Newcastle-upon-Tyne, UK; i Department of Medical Oncology, The Christie NHS Foundation Trust, Manchester, UK; j Department of Oncology, Newcastle Hospitals NHS Foundation Trust, Newcastle-upon-Tyne, UK; k Department of Oncology, Bristol Haematology and Oncology Centre, Bristol, UK; l Department of Clinical Oncology, Weston Park Hospital, Sheffield, UK; m Department of Oncology, Freeman Hospital, Newcastle-upon-Tyne, UK; n Centre for Surgical Research, School of Social and Community Medicine, University of Bristol, Bristol, UK

## Abstract

**Background:**

Peri-operative chemotherapy and surgery is a standard of care for patients with resectable oesophagogastric adenocarcinoma. Bevacizumab, a monoclonal antibody against VEGF, improves the proportion of patients responding to treatment in advanced gastric cancer. We aimed to assess the safety and efficacy of adding bevacizumab to peri-operative chemotherapy in patients with resectable gastric, oesophagogastric junction, or lower oesophageal adenocarcinoma.

**Methods:**

In this multicentre, randomised, open-label phase 2–3 trial, we recruited patients aged 18 years and older with histologically proven, resectable oesophagogastric adenocarcinoma from 87 UK hospitals and cancer centres. We randomly assigned patients 1:1 to receive peri-operative epirubicin, cisplatin, and capecitabine chemotherapy or chemotherapy plus bevacizumab, in addition to surgery. Patients in the control group (chemotherapy alone) received three pre-operative and three post-operative cycles of epirubicin, cisplatin, and capecitabine chemotherapy: 50 mg/m^2^ epirubicin and 60 mg/m^2^ cisplatin on day 1 and 1250 mg/m^2^ oral capecitabine on days 1–21. Patients in the investigational group received the same treatment as the control group plus 7·5 mg/kg intravenous bevacizumab on day 1 of every cycle of chemotherapy and for six further doses once every 21 days following chemotherapy, as maintenance treatment. Randomisation was done by means of a telephone call to the Medical Research Council Clinical Trials Unit, where staff used a computer programme that implemented a minimisation algorithm with a random element to establish the allocation for the patient at the point of randomisation. Patients were stratified by chemotherapy centre, site of tumour, and tumour stage. The primary outcome for the phase 3 stage of the trial was overall survival (defined as the time from randomisation until death from any cause), analysed in the intention-to-treat population. Here, we report the primary analysis results of the trial; all patients have completed treatment and the required number of primary outcome events has been reached. This study is registered as an International Standard Randomised Controlled Trial, number ISRCTN 46020948, and with ClinicalTrials.gov, number NCT00450203.

**Findings:**

Between Oct 31, 2007, and March 25, 2014, 1063 patients were enrolled and randomly assigned to receive chemotherapy alone (n=533) or chemotherapy plus bevacizumab (n=530). At the time of analysis, 508 deaths were recorded (248 in the chemotherapy alone group and 260 in the chemotherapy plus bevacizumab group). 3-year overall survival was 50·3% (95% CI 45·5–54·9) in the chemotherapy alone group and 48·1% (43·2–52·7) in the chemotherapy plus bevacizumab group (hazard ratio [HR] 1·08, 95% CI 0·91–1·29; p=0·36). Apart from neutropenia no other toxic effects were reported at grade 3 or worse severity in more than 10% of patients in either group. Wound healing complications were more prevalent in the bevacizumab group, occurring in 53 (12%) patients in this group compared with 33 (7%) patients in the chemotherapy alone group. In patients who underwent oesophagogastrectomy, post-operative anastomotic leak rates were higher in the chemotherapy plus bevacizumab group (23 [10%] of 233 in the chemotherapy alone group *vs* 52 [24%] of 220 in the chemotherapy plus bevacizumab group); therefore, recruitment of patients with lower oesophageal or junctional tumours planned for an oesophagogastric resection was stopped towards the end of the trial. Serious adverse events for all patients included anastomotic leaks (30 events in chemotherapy alone group *vs* 69 in the chemotherapy plus bevacizumab group), and infections with normal neutrophil count (42 events *vs* 53).

**Interpretation:**

The results of this trial do not provide any evidence for the use of bevacizumab in combination with peri-operative epiribicin, cisplatin, and capecitabine chemotherapy for patients with resectable gastric, oesophagogastric junction, or lower oesophageal adenocarcinoma. Bevacizumab might also be associated with impaired wound healing.

**Funding:**

Cancer Research UK, MRC Clinical Trials Unit at University College London, and F Hoffmann-La Roche Limited.

## Introduction

Randomised controlled trials^1^, ^2^ have shown that the addition of peri-operative chemotherapy to surgery improves survival for patients with resectable oesophagogastric adenocarcinoma compared with surgery alone. Despite this increase in survival, mortality in patients with this disease remains high, with 5-year overall survival for localised disease at diagnosis of only about 40%.^3^, ^4^

Bevacizumab, a monoclonal antibody that targets VEGF, improves responses to chemotherapy and progression-free survival, but not overall survival, in patients with advanced gastric cancer.^5^, ^6^ In oesophagogastric cancer, a complete surgical resection (R0 resection) is an important predictor of long-term survival.^7^ We postulated that a higher proportion of patients responding to pre-operative chemotherapy would increase the likelihood of an R0 resection and lead to improved survival outcomes.^8^ Therefore, we designed ST03 as a phase 2–3 trial to assess the safety and efficacy of adding bevacizumab to peri-operative chemotherapy for patients with resectable oesophagogastric cancer.

The initial phase 2 stage of the trial focused on safety and feasibility in the first 200 patients; these results have been reported previously.^9^ The addition of bevacizumab was feasible and did not seem to significantly increase toxic effects or the likelihood or severity of surgical complications. Therefore, the study proceeded to the phase 3 stage.

## Methods

### Study design and participants

We recruited patients aged 18 years and older with previously untreated, histologically proven, resectable adenocarcinoma of the lower oesophagus, oesophagogastric junction, or stomach from 87 UK hospitals and cancer centres. The original design (from January, 2007) included patients with gastric or Siewert type III oesophagogastric junction tumours, with eligibility widened in July, 2009, to include type II oesophagogastric junction tumours (in response to centres reporting diagnostic difficulty in distinguishing between type II and III oesophagogastric junction tumours), and further widened in March, 2011, to also include type I oesophagogastric junction and lower oesophageal tumours (after the closure of the Medical Research Council [MRC] OE05 trial on Oct 31, 2011), which recruited patients with such tumours). Staging investigations included CT scans for all patients and endoscopic ultrasound for all lower oesophageal and junctional tumours, or according to local practice for gastric tumours. Laparoscopy was mandated for gastric and type II and III oesophagogastric junction tumours, and according to local practice for type I oesophagogastric junction and lower oesophageal cancers. PET scans, MRI, or bone scans were used when clinically indicated according to local practice. To be eligible, patients were required to have a WHO performance status of 0 or 1 and adequate cardiac, liver, renal, and bone marrow function to be eligible. Patients with lower oesophageal or oesophagogastric junction tumours also had to have adequate respiratory function (FEV_1_ ≥1·5 L). Blood pressure of a maximum of 140/90 mmHg, a left ventricular ejection fraction of at least 50%, and the absence of proteinuria were also required.

**Research in context**

**Evidence before this study**

We searched PubMed in April, 2005, for publications relating to bevacizumab in oesophagogastric cancer and bevacizumab in cancer. In 2006, the MAGIC trial showed an improvement in progression-free survival and overall survival when peri-operative chemotherapy was given in addition to surgery, compared with surgery alone, in patients with resectable oesophagogastric adenocarcinoma. In 2011, the AVAGAST trial in advanced gastric cancer reported an improvement in tumour response and progression-free survival, but not overall survival, when bevacizumab was combined with chemotherapy.

**Added value of this study**

To the best of our knowledge, this trial is the first study in which bevacizumab was given to patients with resectable oesophagogastric adenocarcinoma in the peri-operative setting; the results provide no evidence of a benefit of bevacizumab administration in combination with peri-operative chemotherapy in these patients. Moreover, the safety results indicate that bevacizumab administration might also be associated with impaired wound healing.

**Implications of all the available evidence**

The results of this trial suggest that there is unlikely to be a role for bevacizumab in the treatment of localised, operable oesophagogastric cancer. The implication of the results is that patients given standard perioperative chemotherapy are unlikely to benefit from receiving bevacizumab. Future research should consider alternative new treatments in combination with standard therapy in this patient population.

Patients were excluded if they had a medically significant co-existing or previous medical condition, defined as cerebrovascular disease (transient ischaemic attack or stroke), myocardial infarction or angina requiring nitrate therapy within the preceding year, uncontrolled hypertension, a recent history of any gastrointestinal inflammatory disorder or any history of uncontrolled hypertension, congestive heart failure (New York Heart Association grade 2 or worse), or serious cardiac arrhythmia. Those taking corticosteroids or undergoing thrombolytic therapy within 10 days before starting chemotherapy were also ineligible.

All patients gave written informed consent before randomisation. The trial protocol was approved by a national ethics committee and the UK Medicines and Healthcare products Regulatory Agency. Every participating centre obtained local approvals.

### Randomisation and masking

Eligible patients were randomly assigned (1:1) to receive either peri-operative epiribicin, cisplatin, and capecitabine chemotherapy or epiribicin, cisplatin, and capecitabine plus bevacizumab, in addition to surgery. Treatment allocation was done via a telephone call to the Medical Research Council Clinical Trials Unit at University College London (normally by the research nurse at the site who was responsible for following up the patient), where trial management staff used a computer programme that implemented a minimisation algorithm with a random element and stratification by chemotherapy centre, site of tumour (lower oesophagus *vs* oesophagogastric junction type I *vs* type II *vs* type III *vs* stomach), and tumour stage (according to TNM 6th edition). The algorithm established every patient's treatment group allocation at the point of entry (rather than through the use of a pre-determined allocation list). Patients and investigators were not masked to treatment allocation.

### Procedures

Epirubicin, cisplatin, and capecitabine chemotherapy was given as three pre-operative and three post-operative 21-day cycles, consisting of 50 mg/m^2^ intravenous epirubicin and 60 mg/m^2^ cisplatin on day 1 and 1250 mg/m^2^ oral capecitabine on days 1–21. Patients in the chemotherapy plus bevacizumab group were given 7·5 mg/kg bevacizumab as a continuous intravenous infusion on day 1 of each of the chemotherapy cycles (either before or after the chemotherapy was given). To maximise any potential treatment effect with an acceptable toxicity profile, patients in the bevacizumab group also received six further infusions of bevacizumab alone (7·5 mg/kg intravenously alone every 21 days) as maintenance treatment after post-operative chemotherapy.

No bevacizumab dose reductions were allowed. Bevacizumab was discontinued in the event of any new case of gastrointestinal perforation, arterial thromboembolic events (including transient ischaemic attack, stroke, myocardial infarction, or new diagnosis of ischaemic heart disease), grade 3 or 4 haemorrhage, grade 3 or 4 congestive heart failure or left ventricular dysfunction, grade 4 hypertension, grade 4 proteinuria, tracheoesophageal fistula at any grade or any other fistula deemed to be possibly related to bevacizumab. These events were classified as notable, and subject to expedited reporting together with all other toxic effects meeting the standard definitions of serious adverse events. Dose reductions and interruptions in chemotherapy were permitted according to guidance in the trial protocol. All serious adverse events were reviewed for categorisation and severity by the chief investigator (DC) or trial physicians (ECS, AFO).

Surgery was scheduled 5–6 weeks after the last day of the final pre-operative chemotherapy cycle; therefore, there were at least 8 weeks between the last pre-operative bevacizumab administration and surgery. Surgical procedures were specified as follows; for gastric or Siewert type III oesophagogastric junction tumours either proximal, total, or distal subtotal gastrectomy was recommended with a lymphadenectomy to include as a minimum lymph node stations 1–7 to ensure at least 15 nodes were excised; for Siewert type II oesophagogastric junction tumours, either extended gastrectomy or two-phase oesophago-gastrectomy with a two-field lymphadenectomy; for Siewert type I oesophagogastric junction or lower oesophageal tumours, oesophagogastrectomy with either a two phase right thoraco-abdominal approach or a left thoracoabdominal approach with a two field lymphadenectomy. Minimal access procedures were allowed only in centres that had sufficient experience (at least 20 such procedures done) after review of outcomes and complication rates by surgeons from the Trial Management Group. Pathological evaluation of resected tumour specimens followed guidance that was compliant with the Royal College of Pathologists' dataset for oesophagogastric cancer resections.

We assessed tumour response to pre-operative chemotherapy with Response Evaluation Criteria In Solid Tumors (RECIST) criteria (version 1.0; assessed by CT scan, with laparoscopy, endoscopic ultrasound, or PET scans if clinically indicated) and post-operatively at each centre by pathologists who assessed the resected tumour specimen to establish the extent of resection, margin involvement, extent of lymph node dissection, and Mandard tumour regression grade. Resections were judged to be curative (R0) if the pathologist considered a radical resection had been undertaken and there was no evidence of microscopic residual disease with longitudinal margins (proximal and distal) microscopically clear and there were no viable tumour cells present within 1 mm of the oesophageal circumferential resection margins. Post-operative chemotherapy was started 6–10 weeks after surgery. Patients were followed up every 6 months post-surgery for the first 3 years and every year thereafter until death, or at comparable timepoints if treatment was discontinued early. Cause of death and disease progression events were reported according to local investigator assessment.

We assessed quality-of-life data with the European Organisation for Research and Treatment of Cancer (EORTC) QLQ-C30 and STO22 questionnaires, administered before and after post-operative chemotherapy and twice during the maintenance phase, then every 6 months post-surgery for 3 years and annually thereafter. For patients who were not fit for surgery, quality-of-life questionnaires were carried out at similar timepoints. Analysis of quality of life will be presented in a separate publication.

We assessed cardiac function by echocardiogram or multiple gated acquisition scan at baseline and after pre-operative and post-operative chemotherapy. After completion of the phase 2 stage, left ventricular ejection fraction measurements were done only at baseline. Before every chemotherapy cycle, a full blood count and blood pressure measurements were taken and patients were tested for proteinuria. In February, 2010, a protocol amendment mandated a nadir neutrophil count on day 10 of the first pre-operative chemotherapy cycle with granulocyte colony stimulating factor (GCSF) recommended for all cases of grade 4 neutropenia, and at the investigator's discretion for grade 3 cases, during all subsequent chemotherapy cycles.

### Outcomes

For the phase 3 analysis, the primary outcome measure was overall survival defined as the time from randomisation until death. Secondary outcomes were macroscopic disease-free survival, progression-free survival, response rates to pre-operative chemotherapy and curative (R0) resection rates. All efficacy analyses were done on an intention-to-treat basis.

Disease-free survival was measured from a landmark point, taken to be 6 months from randomisation to allow for any difference in timing of surgery across all patients, to the first occurrence of disease recurrence or death. Patients who had an event before the landmark point and those who had a macroscopically incomplete (R2) resection or no resection were deemed to have had a disease-free survival event at time zero. Progression-free survival was measured from randomisation to the first occurrence of disease recurrence or death; unlike disease-free survival, an R2 resection was not considered an event for this outcome measure. In all survival analyses, patients who had not had the event of interest by the time of analysis were censored at the time they were last followed up.

A RECIST response to pre-operative chemotherapy was defined for this trial as a partial or complete response. Those with stable disease, progressive disease, or who had died before the RECIST assessment were regarded as non-responders. For the analysis of pathological tumour response, a Mandard tumour regression grade of 1, 2, or 3 was considered a response and in the intention-to-treat comparison those who did not undergo a resection were included as non-responders.

Sensitivity analysis of overall survival was repeated on the following pre-defined baseline subgroups: age (<60 years; 60–70 years; >70 years); sex; WHO performance status; baseline tumour site; baseline tumour stage (separately for gastric/type III oesophagogastric junction tumours and type I/II oesophagogastric junction/lower oesophageal tumours). We did not do an analysis by the type of surgery because this was not known at baseline; instead we analysed tumour site as a baseline surrogate for this variable.

### Statistical analysis

5-year overall survival in the epiribicin, cisplatin, and capecitabine chemotherapy alone group was estimated to be 40%. This estimate was based on the proportion of patients in the peri-operative chemotherapy group of the MAGIC trial who were alive at 5 years (36%),^1^ taking into consideration the possible effect of improvements in surgical technique, staging, and supportive care over time. A 10% improvement in survival would have been consistent with the benefit seen when adding bevacizumab in other settings at the time the trial was designed.^10^ To detect an absolute 10% improvement in 5-year survival (corresponding hazard ratio [HR] 0·76), with 80% power and a two-sided 5% significance level, 420 deaths were required. On the assumption that the trial would take 3–4 years to complete recruitment, with 18–24 months' follow-up, the target sample size was estimated to be between 900 and 1100 patients. The trial database was frozen for analysis on Sept 30, 2015, after the target number of deaths had occurred. The accumulating data were monitored by an Independent Data Monitoring Committee (IDMC), which met 13 times between May, 2008, and November, 2014, to review safety data and efficacy analyses.

Analyses of survival data were done with the log-rank test. Analyses of overall survival, disease-free survival, and progression-free survival were based on all randomly assigned patients, whereas analysis of overall survival by resection status and Mandard tumour regression grade were based on all randomly assigned patients with available pathological resection status and Mandard tumour regression grade, respectively. We assessed consistency of treatment effect across pre-defined subgroups with tests for heterogeneity that were based on all randomly assigned patients within each subgroup. To compare the two groups in terms of the proportions of patients responding to chemotherapy and the proportions in whom curative resection was achieved, we used the χ^2^ test based on all randomly assigned patients who had available data from the relevant assessment. Comparison of curative resections and pathological responses were repeated on only those patients with available data who underwent a resection. All analyses were unadjusted for covariates and done at a two-sided 5% significance level, with no adjustment for multiple comparisons.^11^ All statistical analyses were done with STATA (version 13.0).

This trial is registered as an International Standard Randomised Controlled Trial, number 46020948, and with ClinicalTrials.gov, number NCT00450203.

### Role of the funding source

Cancer Research UK reviewed and approved the study design. F Hoffmann-La Roche did a factual accuracy check on the final article but any decision to incorporate comments was made solely at the discretion of the authors. Neither funder had any role in the collection, analysis, or interpretation of the data. DC, SPS, ECS, SR, and REL had access to the raw data. The corresponding author had full access to all the data and the final responsibility to submit for publication.

## Results

Between Oct 31, 2007, and March 25, 2014, 1063 patients were enrolled and randomly assigned to receive peri-operative epiribicin, cisplatin, and capecitabine chemotherapy (n=533) or peri-operative chemotherapy plus bevacizumab (n=530). The baseline characteristics were well balanced between the two groups (table 1). The median age of all enrolled patients was 63 years (IQR 56–68), 859 (81%) of 1063 patients were men, and 646 (61%) had stage 3 or 4 disease (according to TNM 6th edition). 144 (14%) had lower oesophageal tumours, 128 (12%) Siewert type I, 199 (19%) Siewert type II, 209 (20%) Siewert type III, and 383 (36%) gastric. The proportion of patients undergoing PET scanning as part of staging has increased steadily during the course of the trial (table 1), but did not appear to differ between the groups.

**Figure 1:**
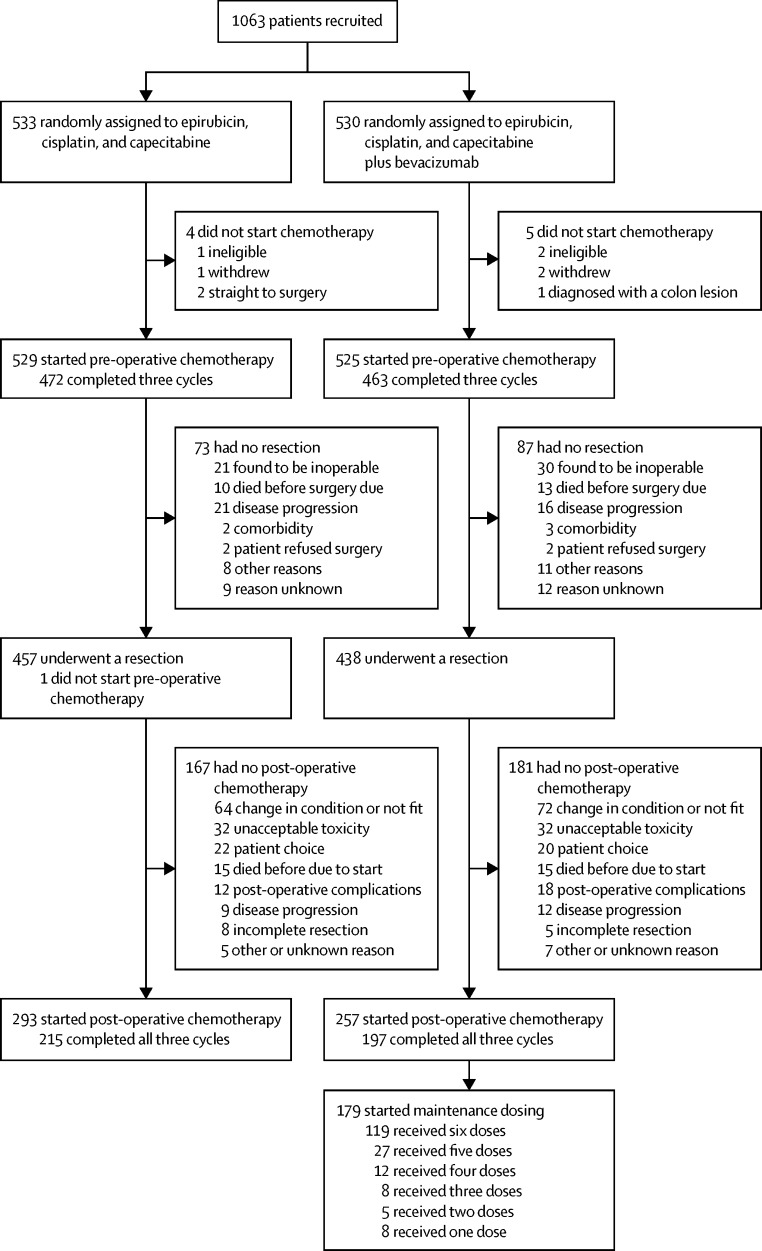
Trial profile

1054 (99%) of 1063 patients (529 in the chemotherapy alone group and 525 in the chemotherapy plus bevacizumab group) started chemotherapy after randomisation (figure 1). 472 (89%) of 529 patients in the chemotherapy group and 463 (88%) of 525 in the chemotherapy plus bevacizumab group who started chemotherapy received all three pre-operative cycles. 895 (84%) of 1063 randomly assigned patients (457 [86%] of 533 in the chemotherapy alone group *vs* 438 [83%] of 530 in the chemotherapy plus bevacizumab group) underwent a resection in the trial. Figure 1 provides reasons why the remaining patients did not undergo surgical resection. The median time from the start of the last pre-operative cycle to surgery was 62 days (IQR 56–68) in the chemotherapy alone group and 62 days (57–69) in the chemotherapy plus bevacizumab group. 843 (95%) of 895 patients had at least 7 weeks between the start of their final pre-operative cycle and surgery. 293 (55%) of 533 patients randomly assigned in the chemotherapy group and 257 (48%) of 530 randomly assigned in the chemotherapy plus bevacizumab group re-commenced chemotherapy post-operatively; 215 (73%) of 293 patients in the chemotherapy group and 197 (77%) of 257 patients in the chemotherapy and bevacizumab group received all three post-operative cycles (figure 1). Of all randomly assigned patients, 212 (40%) of 533 in the chemotherapy alone group and 195 (37%) of 530 in the chemotherapy and bevacizumab group received all six scheduled cycles of chemotherapy. Bevacizumab was given in 1407 (94%) of 1492 cycles administered pre-operatively and 605 (89%) of 679 administered post-operatively. The appendix provides further details about pre-operative and post-operative chemotherapy, including numbers of patients who discontinued treatment or had dose reductions (appendix pp 4, 5).

**Figure 2:**
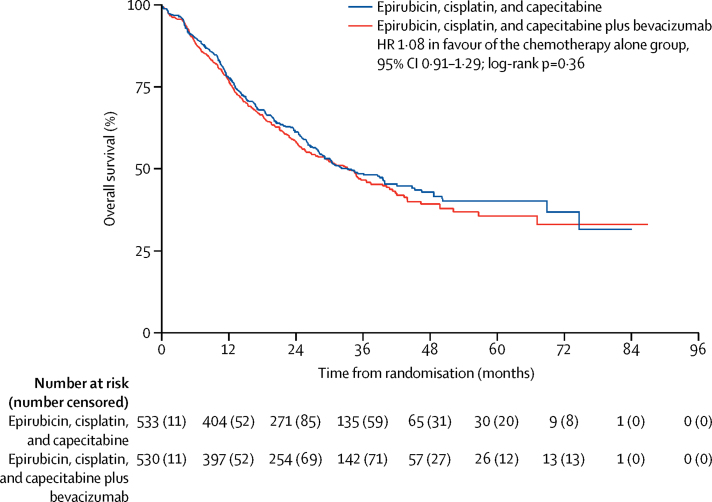
Kaplan-Meier plot of overall survival HR=hazard ratio. Patients still alive at the time of analysis were censored at the time they were last followed up. Survival curves are unadjusted for covariates and the analysis includes all randomly assigned patients.

At the time of analysis, we used a reverse Kaplan-Meier method to calculate median follow-up, which was 38·4 months (IQR 27·5–50·8) in the whole population and 36·2 months (27·4–51·4) in the chemotherapy alone group and 39·1 months (27·6–50·5) in the chemotherapy and bevacizumab group. 508 patients died (248 in the chemotherapy group and 260 in the chemotherapy and bevacizumab group) and 85% of patients in each group (451 of 533 given chemotherapy alone and 452 of 530 given chemotherapy and bevacizumab) had either died or been followed up for at least 2 years. 3-year overall survival was similar in the two groups: 50·3% (95% CI 45·5–54·9) in the chemotherapy alone group and 48·1% (43·2–52·7) in the chemotherapy and bevacizumab group (HR 1·09 in favour of the chemotherapy alone group, 95% CI 0·91–1·29; log-rank p=0·36; figure 2). Insufficient patients (n=56) had reached the 5-year timepoint at the time of analysis to give a reliable estimate of 5-year overall survival.

We recorded 300 disease-free survival events in the chemotherapy alone group and 303 in the chemotherapy plus bevacizumab group. Disease recurrence was confirmed in 210 and 192 patients, respectively (170 and 159 of whom subsequently died), with the remaining events attributable to death before reported recurrence (78 in the chemotherapy group and 101 in the chemotherapy plus bevacizumab group) and a macroscopically incomplete resection (12 *vs* 10). For progression-free survival, a macroscopically incomplete resection was not considered an event of interest, so the total number of events was therefore 288 in the chemotherapy alone group and 293 in the chemotherapy plus bevacizumab group. There was no evidence of a treatment effect of bevacizumab on either disease-free survival (HR 1·04, 95% CI 0·89–1·22; p=0·62) or progression-free survival (HR 1·05, 95% CI 0·89–1·23; p=0·56).

Analysis of RECIST responses to pre-operative chemotherapy (partial or complete response *vs* stable disease, progressive disease, or death before the tumour assessment) is based on 875 patients (438 in the chemotherapy alone group and 437 in the chemotherapy plus bevacizumab group); we excluded 188 patients (95 in the chemotherapy alone group and 93 in the chemotherapy plus bevacizumab group) with missing response data from the pre-operative tumour assessment. The proportion of patients responding to treatment according to RECIST were similar in the two groups (183 [42%] of 438 patients in the chemotherapy group *vs* 177 [41%] of 437 in the chemotherapy and bevacizumab group; p=0·70). Analysis of pathological tumour responses based on Mandard tumour regression grade (grade 1–3 *vs* grade 4–5 or no resection) includes 895 patients (452 chemotherapy alone, 443 chemotherapy plus bevacizumab); those excluded are those who underwent a resection but had unavailable pathological tumour assessment data. The proportion of patients achieving pathological tumour responses were also similar between the groups (147 [33%] of 452 patients in the chemotherapy alone group *vs* 135 [30%] of 443 in the chemotherapy plus bevacizumab group; p=0·51). A repeat of this comparison including only the 727 patients with available data who underwent a resection (376 in the chemotherapy alone group and 351 in the chemotherapy plus bevacizumab group) also yielded a similar result (147 [39%] of 376 patients in the chemotherapy alone group *vs* 135 [38%] of 351 in the chemotherapy plus bevacizumab group; p=0·86).

Comparison of the proportion of resections that achieved R0 is based on 1002 patients (505 in the chemotherapy alone group and 497 in the chemotherapy and bevacizumab group); we excluded 61 patients (28 in the chemotherapy alone group and 33 in the chemotherapy plus bevacizumab group) who underwent a resection but had unavailable pathological assessment data. Resections were judged to be R0 by local pathologists in 321 (64%) of 505 patients in the chemotherapy alone group and 305 (61%) of 497 in the chemotherapy plus bevacizumab group (p=0·47). When the comparison was repeated including only patients who underwent a resection (R0 *vs* R1), the proportions of R0 resections were again similar between the groups (321 [75%] of 429 patients in the chemotherapy alone group *vs* 305 [75%] of 405 in the chemotherapy plus bevacizumab group). Post-hoc, the proportion of R0 resections varied by baseline tumour site; gastric tumours had the highest proportion of R0 resections (265 [87%] of 304 resections), compared with type III oesophagogastric junction (117 [75%] of 157), type II oesophagogastric junction (106 [72%] of 148), type I oesophagogastric junction (62 [61%] of 102), and lower oesophageal (76 [66%] of 116). Of 208 R1 resections, 42 (20%) had a positive proximal margin and 33 (16%) had a positive distal margin (table 2). Circumferential margin involvement was only reported routinely by the pathologist for oesophagogastrectomies; of 146 R1 oesophago-gastrectomies, 72 (49%) had a positive circumferential margin and 132 (90%) were either at or within 1 mm of the circumferential margin.

**Figure 3:**
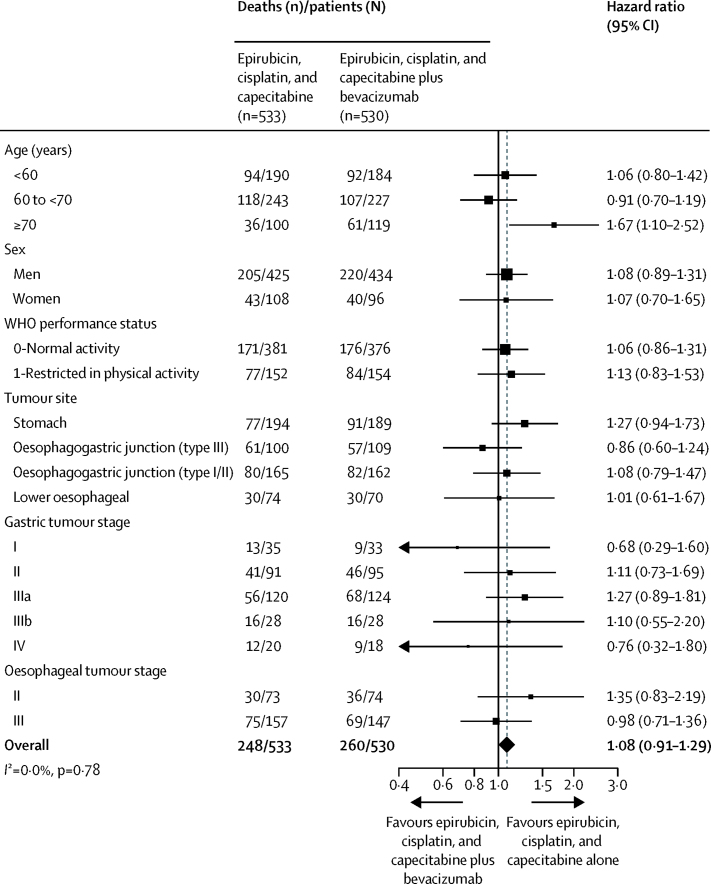
Pre-defined baseline subgroup analysis of overall survival Hazard ratios (HRs) comparing chemotherapy alone with chemotherapy plus bevacizumab in each subgroup are plotted against the horizontal axis, with a HR<1 favouring chemotherapy plus bevacizumab. Black squares represent the HRs, with their size representing the number of patients in the subgroup concerned. Horizontal lines represent 95% CIs for the HRs (arrows indicate that the 95% CI extends beyond the displayed axis range). The diamond in the last row is the overall HR; the vertical dashed line is to aid comparison of the overall HR with the subgroups. None of the four oesophageal stage IVa patients died, so this subgroup is omitted from this figure. 16 patients with type II oesophagogastric junction tumours did not have a baseline oesophageal tumour stage and are also omitted.

Figure 3 shows the results of pre-defined baseline subgroup analyses for overall survival. The results of these analyses were generally consistent with the main result. Although we did not note any heterogeneity overall (*I*^2^=0%, p=0·78) or in most subgroups (sex *I*^2^=0%, p=0·99; WHO performance status *I*^2^=0%, p=0·77; tumour site *I*^2^=0%, p=0·44; gastric tumour stage *I*^2^=0%, p=0·63; oesophageal tumour stage *I*^2^=12%, p=0·29), we did note a trend toward heterogeneity across age groups (*I*^2^=66%; p=0·053). In particular, in patients aged 70 years and older, significantly fewer patients who received chemotherapy alone died compared with those given chemotherapy plus bevacizumab (figure 3). However, in this subgroup, the proportion of deaths that were reported to be non-disease related was higher in the chemotherapy plus bevacizumab group than in the chemotherapy alone group (61 [30%] patients given chemotherapy and bevacixumab *vs* seven [19%] of 36 patients given chemotherapy alone).

**Figure 4:**
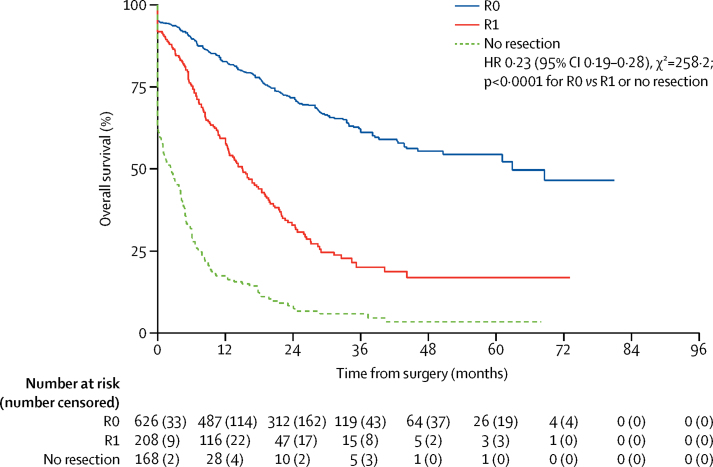
Kaplan-Meier plot of post-operative survival by resection status HR=hazard ratio. Overall post-operative survival times given by the extent of resection, calculated from 6 months post-randomisation until death (to allow for the difference in timing of surgery between the groups). Survival curves are unadjusted for covariates and the analysis includes all patients with non-missing resection outcome data (61 patients with missing data are excluded).

**Figure 5:**
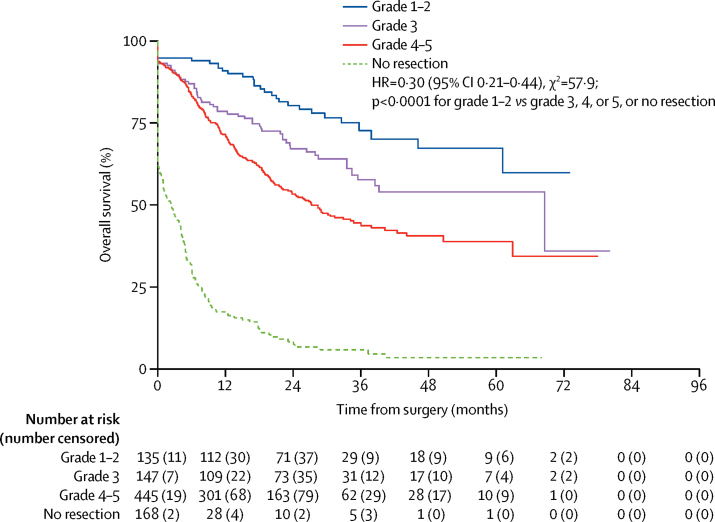
Kaplan-Meier plot of post-operative survival by Mandard tumour regression grade HR=hazard ratio. Overall post-operative survival times given by Mandard tumour regression grade, calculated from 6 months post-randomisation until death (to allow for the difference in timing of surgery between the groups). Survival curves are unadjusted for covariates and the analysis includes all patients with non-missing Mandard tumour regression grade (168 patients with missing data are excluded).

Survival beyond the scheduled surgery timepoint assesed post-hoc was significantly longer in patients who had an R0 resection than in those with an R1 resection or no resection (HR 0·23, 95% CI 0·19–0·28; p<0·0001; figure 4). Our earlier comparison of the proportion of patients achieving a pathological tumour response categorised patients with a Mandard tumour regression grade of 1, 2, or 3 as responders. However, our data suggest that those patients with a Mandard tumour regression grade of 1 or 2 might represent a group with improved post-operative survival (figure 5). When such patients were compared with those with a Mandard tumour regression grade of 3, 4, or 5, or no resection post-hoc, post-operative survival was significantly better (HR 0·30, 95% CI 0·21–0·44; p<0·0001; figure 5).

The frequency and severity of adverse events occurring during either pre-operative or post-operative chemotherapy were similar between the groups (table 3, table 4). Neutropenia was the most common grade 3 or worse adverse event, both pre-operatively (occurring in 145 [27%] of 529 patients in the chemotherapy alone group *vs* 139 [26%] of 525 patients in the chemotherapy plus bevacizumab group) and post-operatively (95 [33%] of 292 *vs* 81 [32%] of 254). This includes two fatal cases of infection with neutropenia, one in the chemotherapy alone group and one in the chemotherapy plus bevacizumab group. Of the 257 patients in the chemotherapy plus bevacizumab group who began post-operative chemotherapy, 179 (69%) went on to receive maintenance bevacizumab and 16 (9%) of these 179 patients reported a grade 3 or 4 toxicity during maintenance treatment, the most common of which were neutropenia (four patients), anorexia (3 patients) and lethargy (3 patients). The most commonly reported serious adverse events were gastrointestinal (60 events in the chemotherapy alone group *vs* 63 in the chemotherapy plus bevacizumab group), anastomotic leaks (30 events *vs* 69), and infections with normal neutrophil count (42 events vs 53).

248 patients in the chemotherapy group and 260 in the chemotherapy plus bevacizumab group had died at the time of analysis. Causes of death were reported to be mainly disease-related (204 [82%] of 248 in the chemotherapy alone group *vs* 204 [78%] of 260 in the chemotherapy plus bevacizimab group); other deaths were due to chemotherapy-related toxic effects (six [2%] *vs* five [2%]), or related to resection or reoperations (13 [5%] in each group). Other reasons were given for 58 patients (23 [9%] in the chemotherapy group *vs* 35 [13%] in the chemotherapy plus bevacizumab group; appendix) and the cause of death was unavailable for the remaining five patients (two [<1%] *vs* three [<1%]).

30-day post-operative mortality was similar in the two groups (14 [3%] of 457 patients who underwent resection in the chemotherapy alone group *vs* 11 [3%] of 438 who underwent resection in the chemotherapy and bevacizumab group). 21 (5%) of 457 patients in the chemotherapy alone group and 22 (5%) of 438 in the chemotherapy and bevacizumab group died within 90 days of surgery. Of the patents who underwent a resection, data from the post-operative assessment were available for 446 patients in the chemotherapy alone group and 427 in the chemotherapy plus bevacizumab group. The overall incidence of post-operative complications was slightly higher in the chemotherapy plus bevacizumab group, with 215 (48%) of 446 patients in the chemotherapy alone group reporting complications compared with 243 (57%) of 427 patients in the chemotherapy plus bevacizumab group. Wound healing complications in particular were more prevalent in the bevacizumab group, occurring in 53 (12%) patients in this group compared with 33 (7%) patients in the chemotherapy alone group. However, the overall incidence of complications that were deemed to be life-threatening was similar in both groups, at 8% (37 of 446 patients in the chemotherapy alone group and 34 of 427 in the chemotherapy plus bevacizumab group). Appendix p 6 provides full details of the post-operative complications.

An increased incidence of post-operative anastomotic leak in the chemotherapy plus bevacizumab group became apparent towards the end of the trial. At a planned IDMC review in June, 2013, leaks were recorded in 30 (10%) of 312 patients in the chemotherapy group and 48 (16%) of 297 in the chemotherapy plus bevacizumab group (compared with 14 [8%] of 179 and 19 [11%] of 170, respectively, at the previous IDMC review in July, 2012). Further investigation showed that the increased leak rate in the bevacizumab group was restricted to those patients who underwent oesophagogastrectomy. In June, 2013, 12 (9%) of 132 patients in this subgroup who received chemotherapy alone had post-operative anastomotic leak versus 29 (24%) of 123 who received bevacizumab, compared with 18 (10%) of 180 versus 19 (11%) of 174, respectively, in all other patients. No other relevant clinical characteristics were identified that might explain the increased frequency of anastomotic leak, nor was there any evidence of a centre effect (data not shown). Consequently, with 1057 patients randomly assigned, recruitment was closed to patients with lower oesophageal or junctional tumours planned for an oesophagogastric resection, and pre-operative bevacizumab was discontinued in such patients who had already been recruited.

At the time of the final analysis (Sept 30, 2015), in patients undergoing oesophago-gastrectomy, we recorded post-operative anastomotic leaks in 23 (10%) of 233 patients in the chemotherapy alone group versus 52 (24%) of 220 in the chemotherapy plus bevacizumab group compared with 20 (9%) of 213 and 23 (11%) of 207, respectively, in all other patients. Overall, most of the 103 cases in which onset dates were available occurred during the period immediately after surgery (40 [39%] within 5 days of surgery and 80 [78%] within 10 days). Leak onset dates were provided on serious adverse event reports and were therefore not available for 15 cases in which the event did not satisfy the criteria for a serious adverse event. In those who had an anastomotic leak, three (7%) of 43 patients in the chemotherapy alone group died within 30 days of the operation versus seven (9%) of 75 in the chemotherapy plus bevacizumab group and revisional operations (appendix) were required in 22 (51%) of 43 patients in the chemotherapy alone group compared with 24 (32%) of 75 in the chemotherapy plus bevacizumab group.

## Discussion

The results of our trial show that the addition of bevacizumab to peri-operative epiribicin, cisplatin, and capecitabine chemotherapy did not improve overall survival in patients with potentially resectable oesophagogastric adenocarcinoma. There was no clinical evidence of a differential biological effect on tumour growth; the proportions of patients responding to pre-operative chemotherapy assessed by both radiological RECIST criteria and Mandard tumour regression grade from the resected specimen, as well as R0 resection rates, were similar in both groups.

This finding is in contrast to those of one small study (n=80), which reported that bevacizumab in combination with docetaxel, oxaliplatin, and fluorouracil increased the proportion of R0 resections achieved in patients with locally advanced gastric cancer,^12^ and results from studies in advanced (unresectable and metastatic) gastric cancer in which bevacizumab in combination with cisplatin and capecitabine given as first-line treatment increased the proportion of patients achieving a response (37·4% *vs* 46·0%; p=0·032) and progression-free survival (HR 0·80; p=0·037) but not overall survival.^5^ Additionally, ramucirumab, a monoclonal antibody against VEGF receptor 2, increases median overall survival compared with placebo from 3·8 months to 5·2 months as second-line treatment in advanced disease^13^ and from 7·4 to 9·6 months compared with placebo, in combination with paclitaxel in the same setting.^14^ Although an insufficient number of patients had reached the 5-year timepoint to give a reliable estimate of 5-year overall survival, the scarcity of evidence for an effect on tumour growth and overall survival to this point suggest that it is unlikely a treatment effect would emerge with longer follow-up.

Neoadjuvant bevacizumab has also been assessed in other tumour types and has been associated with increased clinical and pathological responses;^15^, ^16^, ^17^ however, such an effect was not evident in the ST03 trial. Most (88%) patients in the ST03 trial received 9 weeks of pre-operative chemotherapy, which has previously been shown to enhance tumour down-staging^1^ and improve overall survival. However, the lack of effect shares similarities with results from studies in other tumour types in which promising results with bevacizumab in the advanced setting have not been replicated in earlier stage disease, although these studies were mainly done in the adjuvant setting, for example in breast^18^ and colorectal^19^, ^20^ cancer.

The finding of an increased anastomotic leak rate in patients who had undergone oesophago-gastrectomy was unexpected. The trial was designed such that patients would have at least 8 weeks between their last pre-operative infusion of bevacizumab and surgery. This period extends well beyond the reported half-life of bevacizumab (20 days) and was believed to be sufficient to prevent effects on post-operative outcomes. The primary outcome measures for the phase 2 component were based on tumour perforation rates, cardiac assessments, and post-operative complications.^11^ In the phase 2 analysis (n=200; 101 patients in the chemotherapy alone group, 99 in the chemotherapy plus bevacizumab group), the anastomotic leak rate was 4% in both groups (five cases in each group) with 107 (54%) patients having gastric tumours, 71 (36%) oesophagogastric junction type III, and 22 (11%) oesophagogastric junction type II. These figures compare to 275 (32%), 141 (16%), and 175 (21%), respectively in the subsequent 863 patients, with the remainder having oesophagogastric junction type I (128 patients) and lower oesophageal (144 patients) tumours (recruited after March, 2011). This change in eligibility criteria increased the proportion of patients undergoing oesophago-gastrectomy, potentially explaining why the increased leak rate was not apparent earlier in the trial.

The treatment of anastomotic leaks varies across the UK; however, centres in this study used the same treatment irrespective of which group the patient was in. Surgeons used omentum for anastomosis coverage according to standard practice, although at the time of designing the study, randomised data were not available to support this practice. In this trial, leak was reported by unblinded assessors and clinical, radiological, and surgical definitions were all included. Although there is a possibility that this method overdiagnosed even small (non-clinically significant) leaks, we do not believe that this had a differential effect on rates in each group.

Careful review of possible confounding factors including centre and laparoscopic surgical approaches did not provide any clear explanation for the increased leak rate and suggests that there could be a prolonged effect of bevacizumab that impairs wound healing. Findings of one study^21^ showed that bevacizumab has sustained effects on VEGF inhibition more than 6 weeks after dosing, and findings of several rectal cancer trials in which bevacizumab was used in conjunction with neoadjuvant chemotherapy or chemoradiotherapy showed increased rates of post-operative complications.^22^, ^23^, ^24^, ^25^ Tumour and blood specimens were collected at baseline in this trial and will be used to investigate whether patients susceptible to long-term effects of bevacizumab such as impaired wound healing can be identified.

The potential limitations of our study were the inclusion of both gastric and oesophageal tumours and a generous targeted difference of 10% absolute difference in survival. However, in our subgroup analysis we recorded no indication of a differential effect by tumour site, or any evidence of a differential biological effect based on R0 resection rates, disease-free survival, or progression-free survival. As with the MAGIC trial^1^ that compared surgery alone with surgery and peri-operative chemotherapy, about half of patients (550 [52%] of 1063) started post-operative chemotherapy and only 119 (22%) of 530 in the chemotherapy plus bevacizumab group completed all chemotherapy cycles plus the six cycles of maintenance bevacizumab.

Our data are consistent with findings that R0 resection is an important predictor of long-term survival. In future clinical trials, identification of patients at risk of a positive resection margin might have a role in treatment selection; careful consideration of the clinicopathological features associated with R1 resection will help to inform these decisions. The suggestion from these results that a Mandard grade of 1 or 2 predicts a better survival outcome (as opposed to the usual approach of considering Mandard grades 1–3 as a response and grades 4–5 as no response) requires further evaluation; however, in this trial, patients with a Mandard grade 1 or 2 seem to have improved survival compared with those with grade 3. A similar survival advantage for Mandard grade 1 and 2 responses was seen in the MAGIC trial^26^ and in the recently reported MRC OE05 trial^27^ in which two cycles of neoadjuvant cisplatin and docetaxel, oxaliplatin, and fluorouracil were compared against four cycles of neoadjuvant epirubicin, cisplatin, and capecitabine for oesophagogastric junction and oesophageal adenocarcinoma. However, as intensification of chemotherapy in the OE05 trial did not lead to improved overall survival for the group of patients treated with chemotherapy as a whole, it is unclear whether Mandard tumour regression grade 1–2 (or complete pathological response) is a valid surrogate for overall survival as an endpoint in clinical trials.

In conclusion, the addition of bevacizumab to peri-operative chemotherapy for patients with resectable oesophagogastric cancer did not lead to an overall survival benefit; therefore these results are not practice changing. Ongoing exploration of novel therapies in order to improve outcomes for oesophagogastric cancer patients is warranted.

## Figures and Tables

**Table 1:** Baseline characteristics

		**Epiribicin, cisplatin, and capecitabine chemotherapy alone (n=533)**	**Epiribicin, cisplatin, and capecitabine chemotherapy plus bevacizumab (n=530)**
**Sex**			
Men		425 (80%)	434 (82%)
Women		108 (20%)	96 (18%)
**Age (years)**			
Median (IQR); range		63 (56–68); 31–79	64 (56–69); 28–82
**WHO performance status**			
0 – Normal activity		381 (71%)	377 (71%)
1 – Restricted in physical activity		152 (29%)	153 (29%)
**Pre-treatment tumour site**			
Lower oesophageal		74 (14%)	70 (13%)
Oesophagogastric junction (type I)		62 (12%)	66 (12%)
Oesophagogastric junction (type II)		103 (19%)	96 (18%)
Oesophagogastric junction (type III)		100 (19%)	109 (21%)
Stomach		194 (36%)	189 (36%)
Pre-treatment tumour staging*			
Lower oesophageal, type I or II			
	2a	42 (8%)	43 (8%)
	2b	31 (6%)	31 (6%)
	3	157 (29%)	147 (28%)
	4a†	3 (<1%)	1 (<1%)
	Type II staged as gastric/type III‡	6 (1%)	10 (2%)
Gastric and type III			
	1b	35 (7%)	33 (6%)
	2	91 (17%)	95 (18%)
	3a	120 (23%)	124 (23%)
	3b	28 (5%)	28 (5%)
	4§	20 (4%)	18 (3%)
**Patients undergoing PET scans as part of staging**			
2007–08		5/24 (21%)	4/20 (20%)
2009–10		70/137 (51%)	74/138 (54%)
2011–12		171/263 (65%)	169/259 (65%)
2013–14		81/103 (79%)	82/103 (80%)
**Surgical procedure**			
Lower oesophageal, type I or II			
	Oesophago-gastrectomy	190/239 (79%)	171/232 (74%)
	Total gastrectomy	15/239 (6%)	9/232 (4%)
	Subtotal gastrectomy	0	0
	Distal gastrectomy	1/239 (<1%)	0
	Other or unknown	7/239 (3%)	11/232 (5%)
	No resection	26/239 (11%)	41/232 (18%)
Gastric and type III oesophagogastric			
	Oesophago-gastrectomy	45/294 (15%)	53/298 (18%)
	Total gastrectomy	127/294 (43%)	121/298 (41%)
	Subtotal gastrectomy	16/294 (5%)	17/298 (6%)
	Distal gastrectomy	43/294 (15%)	44/298 (15%)
	Other or unknown	13/294 (4%)	12/298 (4%)
	No resection	50/294 (17%)	51/298 (17%)

Data are n (%), median (IQR; range), or n/N (%).

* Assessed by Tumour Node Metastases 6th edition.

† Stage 4a patients: T2 N1 M1a (one in the chemotherapy alone group); T3 N0–N1 M1a (two in the chemotherapy alone group and one in the chemotherapy plus bevacizumab group).

‡ These patients were randomly assigned after the inclusion of type II oesophagogastric junction tumours but before oesophageal tumour staging was added to case report forms and were therefore staged under the gastric staging system.

§ Stage 4 patients: all T4 N1–N2 M0.

**Table 2:** Surgical and pathological findings

	**Chemotherapy alone (n=533)**	**Chemotherapy plus bevacizumab (n=530)**
**Pre-operative RECIST response**		
Complete response	21/438 (5%)	11/437 (3%)
Partial response	162/438 (37%)	166/437 (38%)
Stable disease	224/438 (51%)	228/437 (52%)
Progressive disease	21/438 (5%)	21/437 (5%)
Died before assessment	10/438 (2%)	11/437 (3%)
Unavailable	95	93
**Extent of resection (pathologist's assessment)**		
R0	321/429 (64%)	305/405 (61%)
R1	108/429 (21%)	100/405 (20%)
No resection	76/429 (15%)	92/405(19%)
Unavailable	28	33
Involved margins*, all resections		
R1	108	100
Proximal margin	24	18
Distal margin	17	16
**Oesophagogastrectomy only**		
R1	75	71
At circumferential margin	43	30
Within 1 mm of circumferential margin	43	43
Either at or within 1 mm of circumferential margin†	69	63
**Lymph node dissection**		
<15 nodes	79/432 (18%)	62/406 (15%)
15–24 nodes	146/432 (34%)	137/406 (34%)
≥25 nodes	207/432 (48%)	207/406 (51%)
No resection	76	92
Unavailable	25	32
**Mandard tumour regression grade**		
Grade 1	30/376 (8%)	37/351 (11%)
Grade 2	38/376 (10%)	30/351 (9%)
Grade 3	79/376 (21%)	68/351 (19%)
Grade 4	128/376 (34%)	115/351 (33%)
Grade 5	101/376 (27%)	101/351 (29%)
No resection	76	92
Unavailable	81	87

Data are n or n/N (%). Percentages are based on all patients with non-missing data; in the summary of lymph node dissection and Mandard tumour regression grade, percentages are based on patients with non-missing data who underwent a resection only.

* Multiple positive margins for an R1 resection might be indicated for a given patient.

† Includes those patients in which a viable tumour was present either at the circumferential margin or within 1 mm of the circumferential margin (the above categories combined).

**Table 3:** Adverse events reported during pre-operative chemotherapy

	**Patients given epiribicin, cisplatin, and capecitabine chemotherapy alone**					**Patients given epiribicin, cisplatin, and capecitabine chemotherapy plus bevacizumab**				
	Total patients	Grade 1–2	Grade 3	Grade 4	Grade 5	Total patients	Grade 1–2	Grade 3	Grade 4	Grade 5
Lethargy	529	373 (71%)	40 (8%)	3 (<1%)	0	525	372 (71%)	38 (7%)	3 (<1%)	0
Nausea	529	317 (60%)	36 (7%)	1 (<1%)	0	525	303 (58%)	23 (4%)	0	0
Alopecia	529	324 (61%)	0	0	0	525	328 (62%)	2 (<1%)	0	0
Neutropenia	529	150 (28%)	114 (22%)	30 (6%)	1 (<1%)	525	150 (29%)	105 (20%)	33 (6%)	1 (<1%)
Palmar-plantar erythrodysesthesia	529	173 (33%)	30 (6%)	0	0	525	178 (34%)	30 (6%)	0	0
Stomatitis	529	174 (33%)	10 (2%)	2 (<1%)	0	525	211 (40%)	10 (2%)	0	0
Vomiting	529	169 (32%)	28 (5%)	1 (<1%)	0	525	157 (30%)	18 (3%)	0	0
Loss of taste	529	168 (32%)	1 (<1%)	0	0	525	184 (35%)	0	2 (<1%)	0
Anorexia	529	148 (28%)	18 (3%)	1 (<1%)	0	525	167 (32%)	18 (3%)	0	0
Diarrhoea	529	134 (25%)	24 (5%)	4 (<1%)	0	525	147 (28%)	27 (5%)	1 (<1%)	1 (<1%)
Peripheral neuropathy	529	92 (17%)	3 (<1%)	0	0	525	94 (18%)	0	0	0
Thrombocytopenia	529	61 (12%)	6 (1%)	0	0	525	66 (13%)	3 (<1%)	2 (<1%)	0
Infection (normal absolute neutrophil count)	529	46 (9%)	6 (1%)	1 (<1%)	0	525	55 (10%)	11 (2%)	0	0
Tinnitus	529	62 (12%)	2 (<1%)	0	0	525	53 (10%)	0	0	0
Hypertension*	477	33 (7%)	0	0	0	468	61 (13%)	4 (<1%)	0	0
Renal toxicity	529	36 (7%)	5 (<1%)	0	1 (<1%)	525	39 (7%)	3 (<1%)	1 (<1%)	1 (<1%)
Infection with neutropenia	529	12 (2%)	18 (3%)	11 (2%)	1 (<1%)	525	15 (3%)	23 (4%)	2 (<1%)	2 (<1%)
Liver toxicity	529	26 (5%)	1 (<1%)	0	0	525	23 (4%)	2 (<1%)	0	0
Pulmonary embolism*	477	1 (<1%)	1 (<1%)	17 (4%)	0	468	0	3 (<1%)	18 (4%)	0
Neurotoxicity	529	21 (4%)	2 (<1%)	0	0	525	18 (3%)	1 (<1%)	0	0
Ototoxicity	529	17 (3%)	1 (<1%)	0	0	525	20 (4%)	0	0	0
Chest pain	529	12 (2%)	2 (<1%)	1 (<1%)	0	525	15 (3%)	5 (<1%)	0	0
Deep vein thrombosis*	477	6 (1%)	4 (<1%)	0	0	468	8 (2%)	11 (2%)	1 (<1%)	0
Haemorrhage*	477	5 (1%)	1 (<1%)	1 (<1%)	0	468	13 (3%)	2 (<1%)	0	0
Arrhythmia*	477	8 (2%)	1 (<1%)	0	0	468	4 (<1%)	1 (<1%)	1 (<1%)	0
Other arterial thromboembolic events*	477	1 (<1%)	1 (<1%)	0	0	468	2 (<1%)	3 (<1%)	2 (<1%)	2 (<1%)
Other venous thromboembolic events*	477	1 (<1%)	0	0	0	468	3 (<1%)	4 (<1%)	2 (<1%)	0
Allergic reaction	529	4 (<1%)	0	0	0	525	4 (<1%)	1 (<1%)	1 (<1%)	0
Myocardial infarction*	477	0	1 (<1%)	2 (<1%)	0	468	0	0	1 (<1%)	1 (<1%)
Cerebrovascular accident*	477	0	0	0	0	468	0	2 (<1%)	0	0
Cardiac failure	529	0	1 (<1%)	0	0	525	0	0	0	0

Table shows all grade 1–2 events occurring in at least 10% patients in either group and all grade 3, 4, and 5 events that occurred. Data are n (%). Events graded according to Common Terminology Criteria for Adverse Events (version 3.0). After each chemotherapy cycle, patients were asked about the occurrence and severity (grade) of several chemotherapy-related toxic effects. These adverse events are presented in order of overall incidence (at any grade), with the most common first.

* These toxic effects were added to the chemotherapy toxicity assessment case report forms after the trial started and as such, not all participants were asked about these specific toxic effects.

**Table 4:** Adverse events reported during post-operative chemotherapy

	**Patients given epiribicin, cisplatin, and capecitabine chemotherapy alone**					**Patients given epiribicin, cisplatin, and capecitabine chemotherapy plus bevacizumab**				
	Total patients	Grade 1–2	Grade 3	Grade 4	Grade 5	Total patients	Grade 1–2	Grade 3	Grade 4	Grade 5
Lethargy	292	204 (70%)	19 (7%)	0	0	254	176 (69%)	24 (9%)	1 (<1%)	0
Nausea	292	186 (64%)	25 (9%)	0	0	254	150 (59%)	14 (6%)	1 (<1%)	0
Neutropenia	292	63 (22%)	73 (25%)	22 (8%)	0	254	54 (21%)	63 (25%)	18 (7%)	0
Diarrhoea	292	124 (42%)	6 (2%)	0	0	254	105 (41%)	7 (3%)	0	0
Alopecia	292	123 (42%)	0	0	0	254	100 (39%)	0	0	0
Anorexia	292	94 (32%)	12 (4%)	0	0	254	89 (35%)	10 (4%)	0	0
Vomiting	292	95 (33%)	14 (5%)	0	0	254	75 (30%)	13 (5%)	1 (<1%)	0
Loss of taste	292	84 (29%)	0	0	0	254	75 (30%)	0	0	0
Stomatitis	292	56 (19%)	0	0	0	254	68 (27%)	4 (2%)	0	0
Palmar-plantar erythrodysesthesia	292	64 (22%)	3 (1%)	0	0	254	56 (22%)	3 (1%)	0	0
Peripheral neuropathy	292	54 (18%)	1 (<1%)	0	0	254	46 (18%)	0	0	0
Thrombocytopenia	292	24 (8%)	1 (<1%)	0	0	254	32 (13%)	0	0	0
Tinnitus	292	36 (12%)	1 (<1%)	0	0	254	18 (7%)	0	0	0
Infection (normal absolute neutrophil count)	292	25 (9%)	3 (1%)	0	0	254	19 (7%)	2 (<1%)	1 (<1%)	0
Proteinuria	273	20 (7%)	1 (<1%)	0	0	237	22 (9%)	0	0	0
Renal toxicity	292	8 (3%)	1 (<1%)	0	0	254	19 (7%)	0	0	0
Infection with neutropenia	292	8 (3%)	7 (2%)	0	0	254	5 (2%)	5 (2%)	1 (<1%)	0
Hypertension*	273	5 (2%)	0	0	0	237	15 (6%)	3 (1%)	0	0
Neurotoxicity	292	7 (2%)	1 (<1%)	0	0	254	7 (3%)	0	0	0
Liver toxicity	292	4 (1%)	1 (<1%)	0	0	254	8 (3%)	0	0	0
Ototoxicity	292	7 (2%)	0	0	0	254	3 (1%)	1 (<1%)	0	0
Reduction in left ventricular ejection fraction*	273	4 (1%)	0	0	0	237	4 (2%)	1 (<1%)	0	0
Pulmonary embolism*	273	0	0	5 (2%)	0	237	0	0	3 (1%)	0
Deep vein thrombosis*	273	0	1 (<1%)	0	0	237	3 (1%)	2 (<1%)	0	0
Other venous thromboembolic events*	273	2 (<1%)	0	1 (<1%)	0	237	3 (1%)	0	0	0
Haemorrhage*	273	0	1 (<1%)	0	0	237	4 (2%)	0	0	0
Arrhythmia*	273	1 (<1%)	0	0	0	237	2 (<1%)	1 (<1%)	0	0
Chest pain	292	2 (<1%)	0	0	0	254	1 (<1%)	1 (<1%)	0	0
Other arterial thromboembolic events*	273	0	2 (<1%)	0	0	237	0	0	1 (<1%)	0
Gastrointestinal perforation*	273	0	1 (<1%)	0	0	237	0	1 (<1%)	0	0
Myocardial infarction*	273	0	0	0	0	237	0	0	1 (<1%)	0
Cardiac failure	292	0	0	0	0	254	0	1 (<1%)	0	0

Data are n (%). Table shows all grade 1–2 events occurring in at least 10% patients in either group and all grade 3, 4, and 5 events that occurred. Events graded according to Common Terminology Criteria for Adverse Events (version 3.0). After each chemotherapy cycle, patients were asked about the occurrence and severity (grade) of several chemotherapy-related toxic effects. These adverse events are presented in order of overall incidence (at any grade), with the most common first.

* These toxic effects were added to the chemotherapy toxic effect assessment case report forms after the trial started and as such, not all participants were asked about these specific toxic effects.
