# Supplementary material for: Peri-operative chemotherapy with or without bevacizumab in operable oesophagogastric adenocarcinoma (UK Medical Research Council ST03): primary analysis results of a multicentre, open-label, randomised phase 2–3 trial
Source: Lancet Oncol. 2017 Mar;18(3):357–70. doi: 10.1016/S1470-2045(17)30043-8 (PMC5337626; doi:10.1016/S1470-2045(17)30043-8)
Supplement: Supplementary appendix [file mmc1.pdf]

THE LANCET
Oncology

Supplementary appendix

This appendix formed part of the original submission and has been peer reviewed. We post it as supplied by the authors.

Supplement to: Cunningham D, Stenning SP, Smyth EC, et al. Peri-operative chemotherapy with or without bevacizumab in operable oesophagogastric adenocarcinoma (UK Medical Research Council ST03): primary analysis results of a multicentre, open-label, randomised phase 2–3 trial. *Lancet Oncol* 2017; published online Feb 2. http://dx.doi.org/10.1016/S1470-2045(17)30043-8.

A randomised phase II/III trial of perioperative chemotherapy with or without bevacizumab in operable oesophagogastric adenocarcinoma: Final results from the UK Medical Research Council randomised ST03 trial

Supplementary data

Appendix table 1: Centre Recruitment and Principal Investigator Details

Centre	Principal Investigator	Patients randomised
Christie Hospital	Dr Was Mansoor	48
Bristol Oncology Centre	Dr Stephen Falk	46
Freeman Hospital, Newcastle	Dr Fareeda Coxon	46
Weston Park Hospital	Dr Suzanne Darby	44
Royal Surrey County Hospital	Dr Sebastian Cummins	42
Birmingham Heartlands	Dr Joyce Thompson	41
St James University Hospital	Dr Matthew Seymour	36
Russell's Hall Hospital	Dr David Ferry	30
Norfolk and Norwich University Hospital	Dr Jenny Nobes	29
Leicester Royal Infirmary	Dr Anne Thomas	27
Guy's Hospital	Dr Paul Ross	25
Maidstone Hospital	Dr Justin Waters	25
Queen Elizabeth, Birmingham	Dr Victoria Kunene	25
Royal Marsden Hospital (Sutton)	Prof David Cunningham	25
St Bart's Hospital	Dr Sarah Slater	25
Clatterbridge Centre for Oncology	Dr Adrian Moss	23
University Hospital Coventry and Warwickshire	Dr Sharmila Sothi	20
Royal Marsden Hospital (London)	Prof David Cunningham	19
Aberdeen Royal Infirmary	Dr Russell Petty	18
Huddersfield Royal Infirmary	Dr Jo Dent	17
Velindre Hospital	Dr Tom Crosby	17
Royal Bournemouth Hospital	Dr Tom Geldart	16
University Hospital of North Staffordshire	Dr Fawzi Adab	16
Castle Hill Hospital	Dr Mohan Hingorani	15
Churchill Hospital	Dr Kinnari Patel	14
Nottingham University Hospital	Dr Srinivasan Madhusudan	14
Wexham Park Hospital	Dr Maher Hadaki	14
Belfast City Hospital	Dr Martin Eatock	13
Cumberland Infirmary	Dr Jonathon Nicoll	13
Musgrove Park Hospital	Dr Emma Cattell	13
Queen's Hospital, Romford	Dr Sherif Raouf	13
Royal Berkshire Hospital	Dr Joss Adams	13
Royal United Hospital	Dr Louise Medley	13
Salisbury District Hospital	Dr Tim Iveson	13
St Mary's Hospital, London	Dr Danielle Power	13
Worthing Hospital	Dr Andrew Webb	13
Beatson Oncology Centre	Prof Jeff Evans	12
Bradford Royal Infirmary	Dr Sue Cheeseman	12

Centre	Principal Investigator	Patients randomised
Cheltenham General	Dr Sean Elyan	12
Great Western Hospital	Dr Claire Blesing	10
Addenbrooke's Hospital	Dr Hugo Ford	9
Derriford Hospital	Dr Sarah Pascoe	8
James Paget University Hospital	Dr Ulrike Dernedde	8
Royal Lancaster Infirmary	Dr David Fyfe	8
Scarborough General Hospital	Dr Mohan Hingorani	8
Southampton General Hospital	Dr Tim Iveson	7
Alexandra Hospital, Redditch	Dr Sharmila Sothi	6
Poole Hospital	Dr Richard Osborne	6
Royal Preston Hospital	Dr Muthu Sivaramalingam	6
St George's Hospital	Dr Tim Benepal	6
Torbay Hospital	Dr Nicole Dorey	6
Countess of Chester Hospital	Dr Shaker Abdallah	5
Glan Clwyd Hospital	Dr Angel Garcia-Alonso	5
Gloucestershire Royal Hospital	Dr Sean Elyan	5
Peterborough City Hospital	Dr Karen McAdam	5
Queen Alexandra Hospital	Dr Caroline Archer	5
Hereford County Hospital	Dr Nick Reed	4
Lincoln County Hospital	Dr Zuzanna Stokes	4
Northampton General Hospital	Dr Somnath Mukherjee	4
St Mary's Hospital, Newport	Dr Judith Cave	4
University Hospital Aintree	Dr Helen Neville-Webbe	4
Ysbyty Gwynedd (Bangor Hospital)	Dr Rachel Williams	4
Airedale General Hospital	Dr Sue Cheeseman	3
Broomfield Hospital	Dr Saad Tahir	3
Burnley General Hospital	Dr Ajay Mehta	3
Darent Valley Hospital	Dr Riyaz Shah	3
Dorset County Hospital	Dr Mike Bayne	3
Furness General Hospital	Dr David Fyfe	3
Hairmyres Hospital	Dr Vivienne Maclaren	3
Halton Hospital	Dr Adrian Moss	3
Royal Blackburn Hospital	Dr Ajay Mehta	3
Royal Cornwall Hospital	Dr Richard Ellis	3
Royal Devon and Exeter	Dr Liz Toy	3
Royal Sussex County Hospital	Dr Andrew Webb	3
Worcestershire Royal Hospital	Dr Charles Candish	3
Basingstoke Hospital	Dr Charlotte Rees	2
North Middlesex Hospital	Dr John Bridgewater	2
Stafford General Hospital	Dr Apurna Jegannathen	2
University College London Hospital	Dr John Bridgewater	2
Wrexham Maelor Hospital	Dr Simon Gollins	2
Doncaster Royal Infirmary	Dr Jonathan Wadsley	1
Pilgrim Hospital	Dr Zuzanna Stokes	1
Princess Alexandra Hospital	Dr John Bridgewater	1

Centre	Principal Investigator	Patients randomised
Royal Free Hospital	Dr Astrid Mayer	1
Royal Hampshire County Hospital	Dr Luke Nolan	1
Victoria Hospital (Blackpool)	Dr Muthiah Sivaramalingam	1
Weston General Hospital	Dr Serena Hillman	1
TOTAL		1063

Appendix table 2: Pre-operative chemotherapy cycle data

		ECX	ECX+B
Patients starting pre-operative chemotherapy		529	525
Chemotherapy status	Received all 3 cycles	472 (89%)	463 (88%)
	Stopped early	57 (11%)	62 (12%)
Reason for stopping early	n	57	62
	Unacceptable toxicity	28 (49%)	26 (42%)
	Death during treatment	9 (16%)	10 (16%)
	Change in patient's condition	7 (12%)	8 (13%)
	Patient's choice	3 (5%)	2 (3%)
	Tumour progression	1 (2%)	1 (2%)
	Intercurrent illness	0 (0%)	1 (2%)
	Other reason	1 (2%)	3 (5%)
	Reason missing	8 (14%)	11 (18%)
Number of cycles started	1	22 (4%)	20 (4%)
	2	35 (7%)	42 (8%)
	3	472 (89%)	463 (88%)
Number of cycles with bevacizumab	0		2 (<1%)
	1		44 (8%)
	2		74 (14%)
	3		405 (77%)
Number of cycles with dose reductions	0	344 (65%)	319 (61%)
	1	118 (22%)	124 (24%)
	2	58 (11%)	70 (13%)
	3	9 (2%)	12 (2%)

Appendix table 3: Post-operative chemotherapy cycle data

		ECX	ECX+B
Patients starting post-operative chemotherapy		293	257
Chemotherapy status	Received all 3 cycles	215 (73%)	197 (77%)
	Stopped early	78 (27%)	60 (23%)
Reason for stopping early	n	78	60
	Unacceptable toxicity	48 (62%)	30 (50%)
	Patient's choice	15 (19%)	12 (20%)
	Change in patient's condition	8 (10%)	5 (8%)
	Intercurrent illness	3 (4%)	3 (5%)
	Tumour progression	0 (0%)	3 (5%)
	Other reason	3 (4%)	6 (10%)
	Reason missing	1 (1%)	1 (2%)
Number of chemo cycles started	1	36 (12%)	24 (9%)
	2	42 (14%)	34 (13%)
	3	214 (73%)	196 (76%)
	Missing	1 (<1%)	3 (1%)
Number of cycles with bevacizumab	0		25 (10%)
	1		23 (9%)
	2		35 (14%)
	3		171 (67%)
	Missing		3 (1%)
Number of cycles with dose reduction	0	116 (40%)	85 (33%)
	1	76 (26%)	86 (33%)
	2	53 (18%)	53 (21%)
	3	47 (16%)	30 (12%)
	Missing	1 (<1%)	3 (1%)

Appendix table 4: Post-operative complications

All patients who underwent a resection had a post-operative assessment approximately six weeks after surgery where details regarding their recovery from the operation were documented. The presence and severity of the complications listed below was evaluated by the local investigator, including whether each complication was felt to be life-threatening or not.

**Complication**	**ECX (N=446)**			**ECX+B (N=427)**		
	**n**	**%**	**LT**	**n**	**%**	**LT**
**Any complication (maximum severity)**	**215**	**48%**	**37**	**243**	**57%**	**34**
Respiratory tract infection	72	16%	6	71	17%	7
Pleural effusion requiring treatment	50	11%	5	41	10%	6
Wound healing complications	33	7%	3	53	12%	5
Wound infection (superficial)	37	8%	2	38	9%	1
Cardiac complications	23	5%	8	30	7%	6
Respiratory failure	27	6%	13	23	5%	13
Intra-abdominal sepsis	18	4%	7	17	4%	7
Wound infection (deep)	15	3%	3	12	3%	4
Empyema	9	2%	1	18	4%	4
Haemorrhage requiring intervention	13	3%	4	13	3%	7
Pulmonary embolism	7	2%	2	9	2%	1
MRSA (any site)	9	2%	0	6	1%	0
Deep vein thrombosis	3	<1%	0	7	2%	1
Other	109	24%	14	118	28%	18
Anastomotic leak*	43	9%		75	18%	
Revisional operation required	39	9%		37	9%	

n = overall number of complications reported (whether life-threatening or not); % = overall percentage of patients reporting the event in question; LT = number of complications, of those reported, that were deemed to be life-threatening by the local investigator.

* Severity information was not collected for anastomotic leak so the number of events that were deemed to be life-threatening is not available.

Appendix table 5: Other causes of death

Group	Cause of death
Chemotherapy alone	pulmonary embolus DVT stomach carcinoma
Chemotherapy alone	small bowel injunction, oesophageal cancer and diabetes type 2
Chemotherapy alone	Admitted with gastro-oesophago-pulmonary fistula to CCU resulting in recurrent thoracic collections & collections + air leak. Died of chest sepsis & severe respiratory faiilure.
Chemotherapy alone	Cerebral infarction
Chemotherapy alone	Left ventricular failure, Ischaemic heart disease, Surgery for Adenocarcinoma of Stomach
Chemotherapy alone	right anterior cerebral infarct
Chemotherapy alone	cerebral haemorrhage - cause unknown
Chemotherapy alone	Cardiac Arrest
Chemotherapy alone	Pneumonia
Chemotherapy alone	1a Aspiration pneumonia, b small bowel resection. II Metastatic recurrent gastric cancer
Chemotherapy alone	Community acquired pneumonia
Chemotherapy alone	severe bilateral bronchopneumonia &recurrent OGJ
Chemotherapy alone	Pulmonary thromboembolism. Cardiac arrest
Chemotherapy alone	PT progressed 29/04/2013 confirmed by CT
Chemotherapy alone	Recurrance at anastomosis
Chemotherapy alone	Middle Cerebral Artery Stroke
Chemotherapy alone	Liver necrosis secondary cirrhosis as a consequence of serum fatty infiltration (NASH)
Chemotherapy alone	Aspiration Pneumonia
Chemotherapy alone	Chest sepsis + multi-organ failure
Chemotherapy alone	Delayed haemorrhage following surgery
Chemotherapy alone	Multi-organ failure
Chemotherapy alone	Pneumonia
Chemotherapy alone	Pneumonia
Chemotherapy plus bevacizumab	unknown, wait outcome of post mortem
Chemotherapy plus bevacizumab	Not known
Chemotherapy plus bevacizumab	heart attack
Chemotherapy plus bevacizumab	Bronchopneumonia
Chemotherapy plus bevacizumab	Metastatic Melanoma
Chemotherapy plus bevacizumab	Pneumonia, bronchiectasis & COPD + oesophageal cancer
Chemotherapy plus bevacizumab	multiple organ failure, neutropenic sepsis and oesophageal Ca (treated with chemo)
Chemotherapy plus bevacizumab	Myocardial Infarction-probably due to chemotherapy
Chemotherapy plus bevacizumab	Pneumonia
Chemotherapy plus bevacizumab	coronary artery disease
Chemotherapy plus bevacizumab	Coronary artery thrombosis
Chemotherapy plus bevacizumab	eroded aorta
Chemotherapy plus bevacizumab	Pneumonia and oesophageal ca.
Chemotherapy plus bevacizumab	Ischaemic Heart Disease due to Coronary artery thrombosis due to coronary artery arturoma
Chemotherapy plus bevacizumab	Post mortem findings natural findings
Chemotherapy plus bevacizumab	a) Extensive intestinal ischaemia due to antecedent causes b) Internal Intestinal Herniation
Chemotherapy plus bevacizumab	Aspiration pneumonia
Chemotherapy plus bevacizumab	metastatic colorectal cancer
Chemotherapy plus bevacizumab	1a multiorgan failure, 1b thoracic sepsis, 1c oesophageal adenocarcinoma (operated)
Chemotherapy plus bevacizumab	coroners case

Chemotherapy plus bevacizumab	Alcoholic Liver Disease
Chemotherapy plus bevacizumab	cardiac arrest
Chemotherapy plus bevacizumab	Cardiac arrest
Chemotherapy plus bevacizumab	Bronchopneumonia
Chemotherapy plus bevacizumab	Disease related and PE
Chemotherapy plus bevacizumab	pneumonia
Chemotherapy plus bevacizumab	Unknown -sudden collapse. Attended air ambulance. Vomitus and haemetemesis reported. Unsuccessful resuscitation. No autopsy performed.
Chemotherapy plus bevacizumab	Brain stem infarction and frontal lobe haemorrhagic stroke
Chemotherapy plus bevacizumab	New Primary - Glioblastoma
Chemotherapy plus bevacizumab	Septic post op - died 7 days post surgery
Chemotherapy plus bevacizumab	Combined toxic effects of buprenorphine and morphine, Carcinoma of oesophagus
Chemotherapy plus bevacizumab	Acute UGI bleed. Oesophageal ulceration
Chemotherapy plus bevacizumab	Unknown - GP contacted but they didnt know
Chemotherapy plus bevacizumab	bilateral pneumonia secondary to aspiration
Chemotherapy plus bevacizumab	Heart attack

Appendix table 6: Description of revisional operations in patients suffering post-operative anastomotic leak

Group	Description of revisional operation
Chemotherapy alone	laparotomy and washout plus feeding jejunostomy
Chemotherapy alone	anastomic leak
Chemotherapy alone	laparotomy, small bowel and caecalexcision & jejunostomy on 8/12/10
Chemotherapy alone	Laparowtomy closed
Chemotherapy alone	right thoracotomy
Chemotherapy alone	To stop bleeding
Chemotherapy alone	Op1: Division of anastomosis, resection of ischaemic segment of conduit. Op2: Venting gastrotomy & oesophagectomy on 03.08.2012. Op3: Reconstitution of small bowel on 20.08.2012Bottom of Form
Chemotherapy alone	1st thoractomy. 2nd laparotomy
Chemotherapy alone	Missing
Chemotherapy alone	re-laparotomy lavage,
Chemotherapy alone	Laparotomy for anastomastic leak
Chemotherapy alone	Wedge resection of transverse colon
Chemotherapy alone	Hemi hepatectomy - showed cirrhosis & infarction
Chemotherapy alone	thoracoscopy/pouch revision/washout
Chemotherapy alone	Laparoscopy and reduction of transient at hernia and coloplasty. 2. and 3. Closure of abdominal wound dehisense
Chemotherapy alone	Rt Thoracotomy-gastric staple
Chemotherapy alone	Thoracotomy and T-tube
Chemotherapy alone	washant and drains inserted
Chemotherapy alone	To repair anastomotic leak
Chemotherapy alone	Thoractomy
Chemotherapy alone	Missing
Chemotherapy alone	ogd anastomatic repair
Chemotherapy plus bevacizumab	repair of anastamotic leak
Chemotherapy plus bevacizumab	Repair of anastomotic leaks
Chemotherapy plus bevacizumab	Patient had a repair of anastomotic leak via thoracotomy
Chemotherapy plus bevacizumab	STENT INSERTION X2
Chemotherapy plus bevacizumab	Anastomotic leak post oesophagectomy re-do
Chemotherapy plus bevacizumab	Perforation at anastomosis sutured
Chemotherapy plus bevacizumab	thoracotomy & wash out
Chemotherapy plus bevacizumab	Laparoscopy, laparotomy and gastroscopy (29/04/2010) and laparotomy and tracheostomy on 08/05/2010
Chemotherapy plus bevacizumab	Laparotomy + jejunostomy tube
Chemotherapy plus bevacizumab	Thoracotomy, lapratomy venting gastrostomy
Chemotherapy plus bevacizumab	Insertion of oesophageal stent
Chemotherapy plus bevacizumab	Laparotomy,defunctioning stoma and mucus fistula
Chemotherapy plus bevacizumab	washout for abcess and leak
Chemotherapy plus bevacizumab	Anastomotic leak repair
Chemotherapy plus bevacizumab	Re look Right thoracotomy
Chemotherapy plus bevacizumab	Anastomotic dehiscence + displaced jejunostomy
Chemotherapy plus bevacizumab	Re-adjustment of an oesophageal t-tube inserted due to anastomotic leak
Chemotherapy plus bevacizumab	anastomotic Leak
Chemotherapy plus bevacizumab	Segmented transverse colectomy

Chemotherapy plus bevacizumab	Right thoractomy and over sewing anastomotic site
Chemotherapy plus bevacizumab	Right thoracotomy + decortication
Chemotherapy plus bevacizumab	Laparoscopic washout
Chemotherapy plus bevacizumab	thoractomy, pleural lavage, mobilisation of gastric conduit and oesophagus, laparatomy and repatriation of stomach, exploration of left neck with exteriorisation of oesophagus
Chemotherapy plus bevacizumab	Laparotomy

Acknowledgements
We are grateful to the following persons who participated in the design, conduct and management of the ST03 trial.

MRC CTU at UCL staff:
Senior scientist/programme lead: Ruth Langley; Project lead/senior statistician: Sally Stenning; Clinical project managers: Lizzie Armstrong, Laura Farrelly, Michelle Gabriel; Trial managers: Rahela Choudhury, Monica Mascarenhas, Claire Robb, Laura Stevenson, Monica Verma; Trial statisticians: Fay Cafferty, Chetan Mistry, Matthew Nankivell, Sam Rowley, Lindsay Thompson; Data managers: Alanna Brown, Alex Chambers, Matthew Fsadni, Katherine Goodall, Linda Ly, Leah Meaden, Dipa Noor, Sara Smart; Data management services: William Cragg, Carlos Diaz Montana, Brendan Mauger

Trial Management Group:
David Cunningham, William H. Allum, Elizabeth Smyth, Alicia Okines, Heike I. Grabsch, Derek Alderson, Thomas Crosby, Robert Mason, S. Michael Griffin, Kate Sumpter, Jane Blazeby, Angela Riddell, Sue Chua

Independent Data Monitoring Committee:
John Bancewicz, Pippa Corrie, Mike Hallissey, Janet Dunn

Independent Trial Steering Committee:
Chris Parker, Philip Johnson, Robin Rudd, Jeremy Whelan, Daniel Fink

Other advisors/collaborators:
Chris Plummer, Yu Jo Chua

Investigators at participating centres:
Aberdeen Royal Infirmary (Russell Petty, Leslie Samuel, Sharon Armstrong, Judith Crawford, Asa Dahle-Smith, Ken Park);
Addenbrooke's Hospital (Hugo Ford, David Gilligan, Susan Harden, Simon Dwerryhouse, Richard Hardwick, Peter Safranek);
Airedale General Hospital (Simon Brown, Ganesan Jeyasangar, Clara Sentamans);
Alexandra Hospital (Kamalnayan Gupta);
Basingstoke and North Hampshire Hospital (Charlotte Rees, Tony Dhillon, Andrew Jackson, Rasheid Mekki);
Beatson West of Scotland Cancer Centre (Jeffry RJ Evans, Janet Graham, Clinton Ali, Peter Correa, Ashita Waterston, Matthew Forshaw, Grant Fullerton);
Belfast City Hospital (Martin Eatock, Claire Harrison, Robert Harte, Paul Henry, Russell Houston, Richard Park, Colin Purcell, Declan Carey, Andrew Kennedy);
Birmingham Heartlands Hospital (Joyce Thompson, Harish Kumar, Martin Richardson);
Bradford Royal Infirmary (Sue Cheeseman, Jay Gokhale, John May);
Bristol Haematology & Oncology Centre (Stephen Falk);
Bristol Royal Infirmary (Paul Barham, Jane Blazeby);
Broomfield Hospital (Saad Tahir, Gopilakrishnan Srinivasan, Michael Harvey, Sri Kadirkarmanathan);
Castle Hill Hospital (Mohan Hingorani, Rajarshi Roy, Martin Gough, Prashant Jain, David Mitton, Elnazeer Salim);
Cheltenham General Hospital (Sean Elyan);
Christie Hospital (Robert Hawkins, Was Mansoor, Fiona Thistlethwaite);
Churchill Hospital (Kinnari Patel, Nicola Warner, R Marshall, Bruno Sgromo);
Clatterbridge Centre for Oncology (Adrian Moss, David B Smith, Brian Haylock, Rosemary Lord, Julie O'Hagan, Andrew Masters);
Countess of Chester Hospital (Shaker Abdallah, Jim Evans, David Monk);
County Hospital (Caroline Connolly, Apurna Jegannathen);
Cumberland Infirmary (Jonathan Nicoll, Simon Raimes, John Wayman);
Darent Valley Hospital (Jasvinder Kaur, Andrew Visioli, Haythem Ali);
Derriford Hospital (Geoffrey Cogill, Sarah Pascoe, Matt Symonds, Joseph Rahamin, Tim Wheatley);
Doncaster Royal Infirmary (Jonathan Wadsley, Srinivasan Balchandra, George Jacob, Clive Kelty);
Dorset County Hospital (Michael Bayne);
Freeman Hospital (Fareeda Coxon, Philip Atherton, Paula Mulvenna, Kate Sumpter);
Furness General Hospital (Chris Ball);
Glan Clwyd Hospital (Angel Garcia-Alonso);
Gloucestershire Royal Hospital (Hugh Barr, Mark Vipond);

Great Western Hospital (Claire Blesing);
Guy's Hospital (London) (Nick Maisey, Paul Ross, Ruhe Chowdhury, Fangfei Gao, Cinta Hierro, Debra Joseph, David Landau, Sarah Ngan, Abrie Botha);
Hairmyres Hospital (Vivienne Maclaren);
Halton Hospital (Amy Ford);
Hereford County Hospital (Nick Reed);
Huddersfield Royal Infirmary (Jo Dent, Chris Fosker, Uschi Hofmann, Emma Woodward);
James Paget Hospital (Ulrike Dernedde, Zacharias Tasigiannopoulos);
John Radcliffe Hospital (Nick Maynard);
Leicester Royal Infirmary (Anne Thomas, Christopher Sutton);
Lincoln County Hospital (Zuzana Stokes, Thomas Sheehan, David Andrew);
Liverpool Heart and Chest Hospital (Nathan Howes);
Maidstone Hospital (Justin Waters, Sam Chan, Mathilda Cominos, Mark Hill, Emma Kipps, Alicia Okines, Shazza Rehman, Timothy Sevitt, Riyaz Shah, Anand Sharma, Alicia Synowiec, Kiruthikah Thillai, Ahmed Hamouda);
Manchester Royal Infirmary (Alan Li, Robert Pearson);
Musgrove Park Hospital (Emma Cattell, Julie Walther, Hilary Barlow, Erica Beaumont, Mary Tighe);
Norfolk and Norwich University Hospital (Jenny Nobes, Tom Roques);
North Middlesex Hospital (John Bridgewater, Cheng Yeoh);
Northampton General Hospital (Roshan Agarwal, Gerard Andrade, Somnath Mukherjee, Rachel Gabitass);
Nottingham University Hospitals, City Campus (Srinivasan Madhusudan, Eleanor James);
Peterborough City Hospital (Sarah Ayers);
Peterborough District Hospital (Karen McAdam, Catherine Jephcott, Marie Waters, Kamarul Zaki);
Pilgrim Hospital (Alfredo Addeo);
Poole Hospital (Richard Osborne, Shorland Hosking, David Tarver);
Princess Alexandra Hospital (Harlow) (Lucinda Melcher);
Queen Alexandra Hospital (Caroline Archer, Mya Gyi, Kudingila Madhava, Stuart Mercer, Shaw Somers, Simon Toh);
Queen Elizabeth Hospital (Birmingham) (Victoria Kunene, David Peake, Derek Alderson, David Gourevitch, John Whiting);
Queen's Hospital (Romford) (Sherif Raouf, David Khoo, Dipankar Mokherjee);
Rochdale Infirmary (Khurshid Akhtar, Siba Senapati);
Royal Berkshire Hospital (Joss Adams, James Gildersleve, Ruth Davis, Shahid Sharif, Michael Booth, Thomas Dehn);
Royal Blackburn Hospital (Wiebke Appel);
Royal Bournemouth Hospital (Tom Geldart, George Astras, Tamas Hickish, David Bennett, Nick Davies);
Royal Cornwall Hospital (Richard Ellis);
Royal Devon and Exeter Hospital (Elizabeth Toy, Richard Berrisford, Martin Cooper, Saj Wajad, Saj Wajed);
Royal Free Hospital (Astrid Mayer, Roopinder Gillmore);
Royal Hallamshire Hospital (Roger Ackroyd, Chris Stoddard, Andrew Wyman);
Royal Hampshire County Hospital (Luke Nolan, Rao Vuyyuru);
Royal Lancaster Infirmary (David Fyfe);
Royal Liverpool University Hospital (Mark Hartley);
Royal Marsden Hospital (London) (Ian Chau);
Royal Marsden Hospital (Sutton) (David Cunningham, Sheela Rao);
Royal Preston Hospital (Muthu Siva(Sivaramalingam), Ajay Mehta, Elaine Young, Christopher Ball, Paul Turner, Jeremy Ward);
Royal Stoke Hospital (Fawzi Adab, Arshad Jamil, Duncan Beardsmore, Chandra Cheruvu, William Crisp, M Deakin, Damien Durkin);
Royal Surrey County Hospital (Sebastian Cummins, Gary Middleton, Shaun R Preston, Oliver Priest, Yuen Soon);
Royal Sussex County Hospital (Andrew Webb, Rebecca Herbertson, Ruth Langley, Geoff Newman, Angus Robinson, PC Hale, Don Manifold);
Royal United Hospital (Louise Medley, Tania Tillett, Emma De Winton, Abigail Jenner, Susan Masson, Richard Krysztopik);
Royal Victoria Hospital (Belfast) (Barry Clements);
Russells Hall Hospital (David Ferry, Mano Joseph, Muhammed Khan, Philip Earwaker, Lalit Pallan);
Salford Royal Hospital (Laura Formela);
Salisbury District Hospital (Adityanarayan Bhatnagar, Melanie Harvey);
Scarborough General Hospital (Georgios Bozas, Amandeep Dhadda);

Southampton General Hospital (Tim Iveson, Andrew Bateman, Ian Bailey, James Byrne);
St Bartholomews Hospital (London) (David Propper, Sarah Slater, Marco Gerlinger, Gargi Patel, Frances Hughes);
St George's Hospital (London) (Taqdir (Tim) Benepal);
St James University Hospital (Leeds) (Matt Seymour, Alan Anthoney, Daniel Swinson, Simon Dexter, Jeremy Hayden, Abeezar Sarela, Henry Sue-Ling);
St Mary's Hospital (London) (Danielle Power);
St Mary's Hospital (Newport) (Judith Cave, Prokopios Dimopoulos);
St Thomas's Hospital (London) (Robert Mason);
Tameside General Hospital (Abduljalil Benhamida);
Torbay District General Hospital (Nicole Dorey, Rajaguru Srinivasan, Grant Sanders);
University College Hospital (Daniel Hochhauser, Khaled Dawas, Mughal Muntzer);
University Hospital Aintree (Helen Neville-Webbe, Chan Ton, Graeme Poston);
University Hospital Coventry and Warwickshire (Sharmila Sothi, Martin Scott-Brown, Vinod Menon, Lam Chin Tan);
Velindre Hospital (Tom Crosby, Alison Brewster, Carys Morgan);
Victoria Hospital (Blackpool) (Pavel Bezecny);
Warrington Hospital (Martin Brett);
Weston General Hospital (Serena Hilman, Marjorie Tomlinson, Tom Wells);
Weston Park Hospital (Suzanne Darby, Linda Evans, Debra Furniss, Lucy Walkington);
Wexham Park Hospital (Maher Hadaki, Marcia Hall, Catherine Harris, Bob Soin);
Wishaw General Hospital (Hakim Ben Younes);
Worcestershire Royal Hospital (Charlie Candish);
Worthing Hospital (Khaled Hamdan, Krishna Singh);
Wrexham Maelor Hospital (Simon Gollins);
Wythenshawe Hospital (Ian Welch);
Ysbyty Gwynedd (Rachel Williams, Catherine Bale, Claire Fuller, Anna Mullard, Nick Stuart);
